# Treatment Preferences of Residents Assumed to Have Severe Chronic Diseases in China: A Discrete Choice Experiment

**DOI:** 10.3390/ijerph17228420

**Published:** 2020-11-13

**Authors:** Yinghao Lv, Qiang Fu, Xiao Shen, Erping Jia, Xianglin Li, Yingying Peng, Jinghong Yan, Mingzhu Jiang, Juyang Xiong

**Affiliations:** 1 Department of Health Administration, School of Medicine and Health Management, Tongji Medical College, Huazhong University of Science and Technology, Wuhan 430030, China; m201975508@hust.edu.cn (Y.L.); shenxiao0714@hust.edu.cn (X.S.); m201875310@hust.edu.cn (E.J.); m201875311@hust.edu.cn (X.L.); m201875309@hust.edu.cn (Y.P.); m201975510@hust.edu.cn (J.Y.); jiangmingzhu@hust.edu.cn (M.J.); 2 Department of Epidemiology and Biostatistics, College for Public Health and Social Justice, Saint Louis University, St Louis, MO 63103, USA; john.fu@slu.edu

**Keywords:** chronic diseases, treatment preference, discrete choice experiment, care residents, willingness to pay, traditional Chinese medicine

## Abstract

Objectives: This study aims to elicit the relative importance of treatment attributes that influence residents’ choice, assuming they are suffering severe non-communicable diseases (NCDs), to explore how they make trade-offs between these attributes and to estimate the monetary value placed on different attributes and attribute levels. Methods: A discrete choice experiment (DCE) was conducted with adults over 18 years old in China. Preferences were evaluated based on four treatment attributes: care provider, mode of service, distance to practice and cost. A mixed logit model was used to analyze the relative importance of the four attributes and to calculate the willingness to pay (WTP) for a changed attribute level. Results: A total of 93.47% (2019 of 2160) respondents completed valid questionnaires. The WTP results suggested that participants would be willing to pay CNY 822.51 (USD 124.86), CNY 470.54 (USD 71.41) and CNY 68.20 (USD 10.35) for services provided by experts, with integrated traditional Chinese medicine (TCM) and Western medicine (WM) and with a service distance <=30 min, respectively. Conclusions: The results suggested that mode of service, care provider, distance to practice and cost should be considered in priority-setting decisions. The government should strengthen the curative service capability in primary health facilities and give full play to the role of TCM in the prevention and treatment of severe chronic diseases.

## 1. Introduction

Interwoven with an ageing population, behavioral changes [1,2,3] and rapid urbanization [4,5], non-communicable diseases (NCDs), like cardiovascular diseases and hypertension, have been a major threat to public health worldwide. More than 300 million chronically ill patients have been diagnosed in 2018 in China; especially the incidence of cardiovascular and cerebrovascular diseases increased from 28.28% in 2008 to 36.67% in 2012 [6]. At the same time, severe NCDs have shown a trend of development in young adults and have begun to affect middle-aged people [7].

The Chinese government had taken a series of measures to alleviate the threat from NCDs ten years ago. In 2009, China started its health system reform, which aimed to establish an accessible, equitable, affordable and efficient health system to cover all of the population by 2020. The primary healthcare system was designated as a means of addressing the emerging dual burden of NCDs and infectious diseases in the national “Healthy China 2030 plan” [8,9,10]. In 2017, the central government issued “China’s long-term plan for the prevention and treatment of NCDs (2017–2025)”, which focused on controlling risk factors of NCDs [11]. Obviously, these public health policies underlined the governments’ commitment and determination to cope with the challenges raised by NCDs.

With the Chinese government’s efforts, 95% of the population has been covered by social basic medical insurance schemes at the end of 2017 [12]. The per capita fund for each resident based on the national basic medical insurance has increased from CNY 100 (USD 15.18) in 2008 to CNY 700 (USD 106.26) in 2018 [13]. However, the enhanced financing level had not yet met the severe NCDs patients’ demand for primary healthcare in China [14]. Low capability and supply mismatch led to the poor performance of severe NCDs management. In terms of service supply, health care providers had not fully considered the needs of patients, delivering a large number of services that were not needed [15]. A study showed that 30% of medical resources were wasted, nearly 30% of inspections and treatments were unnecessary [16]. While for capability, services of the primary healthcare facilities are considered of low quality in China. Residents are inclined towards seeking better quality services in tertiary hospitals [17]. If the health care providers do not take the patients’ needs and preferences into account, patients probably would not use primary healthcare fully and would continue wasting the limited medical resources.

To allocate and use medical resources effectively, it is important to understand the preferences of residents with severe NCDs. The growing evidence shows that people actively make choices in seeking medical care and highlights the need to understand residents’ preference for choosing health services [18]. Kleij’s research found that people were prepared to tolerate longer waiting time in hospitals with a better reputation and a shorter distance to practice [19]. In China, it has been proven that factors such as operation level, nursing quality and equipment conditions can affect patients’ choices [20]. Some researchers suggested that adjusting medical care reimbursement rates might contribute to attaining patients’ needs [20,21]. It indicates a crucial need to explore the preferences from patients’ perspective. Within the context of the NCDs management in China, this study used a discrete choice experiment (DCE) to explore the treatment preferences of residents assumed to have severe NCDs. Our specific aims are to: (1) assess the medical service utilization preferences of residents (2) calculate the residents’ willingness to pay (WTP) for different attributes of health services, and (3) identify which factors are important and impact residents with severe NCDs’ preferences of health care usage.

## 2. Materials and Methods

DCE is a choice-based variant of conjoint analysis that became useful through the theoretical study of Lancaster [22] and McFadden [23]. The theoretical foundation for DCEs was provided in random utility theory [24], a discrete choice is offered and participants choose the option with the highest utility among the alternatives presented. Choices are then analyzed to deconstruct respondents’ preferences based on the alternatives that they have chosen [25,26]. In this study, we used a DCE, which can be described as five key stages [27,28]: (1) selection of attributes and levels, (2) questionnaire design, (3) pilot testing, (4) data collection, and (5) statistical analyses.

### 2.1. Study Design

#### 2.1.1. Selection of Attributes and Levels

We began the process of attributes selection from three starting points. Firstly, it was the idea that the most important factors for residents with severe NCDs may include the timeline and convenience of seeking medical care. Secondly, residents with severe NCDs may give priority to seeking experts in tertiary hospitals in the context of China’s healthcare system. Thirdly, we also considered the factors related to the cost of medical care.

Then, we determined the attributes which were the most realistic and credible for residents who were assumed to have severe NCDs [29]. Understanding relevant preference of attributes and levels is crucial for designing any stated-preference study [30]. We drew on recent theoretical [31] and empirical research [32] to address this issue and ensure that the DCE was valid and reliable. Furthermore, we conducted the literature review and qualitative studies. Ten experts (six participants who came from hospitals, community health service centers involved with NCDs management and four scholars devoted to health care delivery research) were invited to join the semi-structured interviews in Wuhan City between November and December in 2017. We presented the top six alternative attributes of preference from attributes pool by literature review, each with two or three levels for discussion in the group. The experts reached the consensus based on the results of panel discussion, and we ultimately determined the following top ranked four attributes: (1) care provider, (2) mode of service, (3) cost, and (4) distance to practice.

We used professional terminology, exact wording to describe the attributes, and reasonably defined the level of the attributes. The attributes were designed at 2 or 3 levels each. We described some levels using quantitative way, which is more interpretable. For example, the levels of cost ranged from CNY 100 (USD 15.18) to CNY 300 (USD 45.54), which was based on the 2017 China Statistical Yearbook. Our DCE’s attributes and levels are shown in Table 1.

#### 2.1.2. Questionnaire Design

The combination of attributes and levels resulted in 36 possible scenarios (two attributes at two levels and two attributes at three levels = 2^2^ × 3^2^) and a total of 630 possible pair choices ((36 × 35)/2). We designed the DCE to provide maximal statistical efficiency for a manageable length of questionnaire by applying orthogonal design [34]. It reduced the number of choice scenarios to a practical number of 16 representative combinations using IBM SPSS Statistics (version 22.0) (IBM, Armonk, NY, USA), which ensured balance among the attributes and levels. Since 16 choice sets could be excessively burdensome for respondents to complete in the limited time, the 16 choice sets were divided into two separate blocks of the questionnaire. Each choice has two options labelled as option 1 and option 2 (a sample scenario is shown in Table 2).

We created a scenario to examine the effect of context on decision making. In this scenario, the respondents have a serious illness that seriously affected his or her daily life. Although the treatment could prolong survival time and improve the quality of life, it would not necessarily cure the disease.

To ensure the quality of the data, we provided respondents with a rationality test. We made one of the choice alternatives superior, because the respondents had an a priori tendency toward attribute-level ordering. They were considered to have failed the test if they did not choose the dominant alternative. Apart from the 16 choice tasks previously described, the questionnaire further contained 12 demographic questions.

The final questionnaire consisted of three sections. The first section included the introduction of the background of the survey, followed by the 12 demographic questions. The second part included the rationality tests. The third section presented 8 DCE pairwise choice tasks.

#### 2.1.3. Pilot Testing

To test the quality and feasibility of our questionnaire, we conducted a face-to-face survey of 60 volunteers in a community in Wuhan City. During the interviews, the understanding of the questionnaire, the time to complete the questionnaire and the validity of the questionnaire content were recorded. The respondents needed, on average, 15 min to complete the interview. According to the pre-survey, we made minor adjustments to the content of the questionnaire.

### 2.2. Data Collection

To make the sample representative, the survey was undertaken in six cities (including Pudong in Shanghai, Taizhou in Jiangsu Province, Xuchang in Henan Province, Guiyang in Guizhou Province, Wuhan in Hubei Province, and Chengdu in Sichuan Province) from May to August in 2018. According to the economic development level, the six cities represented the eastern region (Shanghai and Taizhou), central region (Wuhan and Xuchang) and western region (Chengdu and Guizhou). In each city, 6 primary care institutions were selected, including urban (3 community health centers) and rural areas (3 township health centers). A sample of approximately 60 participants was randomly selected from each institution. Respondents were eligible for inclusion in the DCE if they: (1) were over 18 years old; (2) had utilized medical services before; (3) could complete the questionnaire independently; (4) spoke Mandarin.

We contacted the local community health centers and township health centers to get their support before the interview. The respondents were selected randomly from the regional jurisdiction population by random number table methods until the number reached 60 in the target area. We invited them to participate in our survey by phone notification. To improve their survey adherence, the community health centers/township health centers provided them with a free health physical examination. Face-to-face interview was undertaken in 36 primary care institutions. Before commencing the interview, all interviewers received training about a general overview of the study, a detailed review of the questionnaire, an instruction on questionnaire administration and role-playing in the questionnaire-based interview [35].

We administered 2160 questionnaires and received 2019 valid questionnaires with a response rate of 93.47%, which were collected by eliminating extreme values and missing values. The research methods and investigation tools in this study were approved by the Ethics Committee of Tongji Medical College, Huazhong University of Science and Technology (IORG No: IORG0003571).

### 2.3. Statistical Analysis

Data were entered into EpiData 3.1 (EpiData Association, Odense, Denmark) and transferred to Stata 12.0 (Stata Corp LLC, College Station, TX, USA). Descriptive statistics were used to describe participant’s socio-demographic characteristics. DCE data was analyzed via the mixed logit model which can take the potential preference heterogeneity of participants into account. The utility associated with a particular treatment regimen is made up of 2 components: the deterministic component and the unobservable component. Categorical variables (for the attributes of the mode of service, care provider, and distance to practice) were coded as dummy variables while the cost was specified as a continuous variable in the model to facilitate the calculation of WTP, that is, the relative monetary value that residents with severe NCDs placed on various aspects of the treatment options. For analysis, the reference level was traditional Chinese medicine (“TCM”), “≤30 min”, and “general practitioner”. Regression coefficients were estimated for all attributes in the regression model. The magnitude of the regression coefficients represented the degree of preference for each of the attributes, the larger the coefficient was, the more likely it was that attribute was preferred. The ratio of the cost coefficient to other coefficients was used to calculate the WTP for marginal changes in the corresponding attributes [36].

## 3. Results

### 3.1. Characteristics of the Respondents

The numbers of participants from the six cities were 349 (Pudong), 316 (Taizhou), 357 (Xuchang), 406 (Wuhan), 358 (Chengdu), and 374 (Guiyang). In total, 105 participants failed the consistency test. The characteristics of the respondents are presented in Table 3. There were no significant differences between the participants who passed or failed the consistency test. The mean age of the analytical sample (n = 2019) was 47.50 ± 18.11 years, and the average monthly income was CNY 5503.59 ± 865.18 (USD 835.45 ± 131.33). Female participants (67.40%) were overwhelmingly dominant in the sample.

### 3.2. Main Effects Model Results

Table 4 shows the main results of the mixed logit model. The mean coefficients of the four medical service predictors were statistically significant (*p* < 0.01). Standard deviations indicated preference heterogeneity. The SDs of the four coefficients were statistically significant, indicating the presence of significant preference heterogeneity for treatment attributes in this population. Participants considered the mode of service, distance to practice, cost and care provider in priority-setting decisions when receiving treatment services. Among nonmonetary attributes, it is evident that when choosing treatment services, participants assumed to have severe NCDs preferred integrated TCM and Western medicine (WM) services, distance to practice ≤ 30 min and services provided by expert. Participants expressed the highest stated preferences for treatment with “services provided by expert” (β = 0.847, *p* < 0.01), followed by “integrated TCM and WM services” (β = 0.485, *p* < 0.01). The “distance to practice” also had a substantial impact on the utility of participants using treatment services (β = −0.071, *p* < 0.01), which indicated that participants preferred treatment services that were closer to them.

### 3.3. Willingness to Pay

WTP represents a monetary measure of participants’ valuation of a certain attribute. It was the ratio of the coefficient between each attribute level and the cost [25].

The results of the WTP calculation (shown in Table 4) are used for relative comparisons. The WTP for the care provider and mode of service provides a clear indication regarding the importance of these two attributes. As indicated in Table 4, the residents assumed to have severe NCDs were willing to pay CNY 822.51 (USD 124.86) and CNY 470.54 (USD 71.41) to access GP services and obtain integrated TCM and WM services, rather than accessing general services and TCM services. When residents received additional compensation of CNY 68.20 (USD 10.35), they were willing to change the distance to practice from less than 30 min to more than 30 min.

## 4. Discussion

This study identified preferences for treatment attributes among respondents who were assumed to have severe NCDs in China. All four attributes significantly affected the preferences of the participants. Under the assumption of severe chronic disease, participants preferred services provided by experts, services located at a relatively short distance and integrated TCM and WM services. There is a trade-off between the service attributes, in which respondents are willing to pay a certain fee in exchange for an improvement in a certain service attribute. For example, for the mode of service, the residents with severe NCDs are willing to pay an additional CNY 822.51 (USD 124.86) to receive services provided by experts.

According to the DCE result, policy interventions that focus on enhancing accessibility to health care institutions will be an effective approach to attract residents with severe NCDs. Previous studies have found that as NCDs require long-term monitoring and management, residents with severe NCDs prefer to accept services from medical service institutions that are close to them [37]. In this study, the attribute of medical treatments also included the distance to practice, and the residents with severe NCDs were only willing to change the visiting site from less than 30 min to more than 30 min when they received additional compensation. A study shows that the further a resident lives from the health care institutions, the worse their health consequences are [38]. However, with the development of internet technology, telemedicine uses information systems and communication systems can overcome geographical barriers and increase accessibility to health care services. Then, telemedicine can be fully utilized to increase the accessibility and availability of primary health care institutions.

Similar to other studies, our findings implied that when the disease is severe, residents preferred to receive integrated TCM and WM services provided by experts [39]. In addition, Zhang concluded that with the emergence of new disciplines and the transformation of the medical mode, patients’ demands for integrated TCM and WM services is on the rise [40]. Previous studies showed that TCM for prevention and treatment is acknowledged and it has its own unique advantages, such as safety, lower cost, effectiveness [41,42]. In the provision of treatment options for residents with severe NCDs, full consideration of resident’ preferences and needs can guide health care professionals to provide the appropriate types of treatment services for targeted patient groups. Therefore, it is crucial to strengthen the application of integrated TCM and WM services in NCDs treatment. The vigorous development of NCDs management services is inseparable from policy support, which can achieve the goal of harmonizing the diversity of medical care demand. The results indicated that policymakers should fully consider the positive role of integrated TCM and WM services and expert treatment services. Formulating the supporting policy such as increasing financial support, which will bring integrated TCM and WM service’s superiority into full play in primary healthcare facilities, should be a consideration for government.

As for willingness to pay for attributes levels, DCE cannot confirm whether participants would pay these amounts to receive these services. The method only requires respondents to make assumptions rather than actual choices [43]. However, we may infer that individuals may be willing to pay more money for these services when the disease, becomes worse because the patient would feel less sensitivity to more out-of-pocket cost when they are eager to recover soon [44]. Interestingly, the WTP results suggested that the services provided by experts could have a significant impact on health care preferences. The value the respondents attributed to the services provided by experts is equal to CNY 822.51 (USD 124.32). It suggested that respondents had the highest willingness to pay for the services provided by experts, reflecting that they valued the quality of healthcare highly when the disease became worse [45,46]. A notion that has been held in residents is that the expert can provide better quality care than the GP. Hence, it is necessary to formulate the supporting policy such as increasing human resources and encourage experts from higher level hospitals to provide services in the primary health care institutions.

Several limitations should be noted. Firstly, while we selected the most important attributes from the literature review and qualitative studies, other factors may not have been taken into account. Secondly, respondents need to make choices under certain circumstances, and the stated preference may be different from the revealed preference, which is also the limitation of the stating partiality method. It is also unclear whether individuals would pay as much in real life as they stated. This study only analyzes the service utilization preferences under a hypothesis and does not serve the actual sick and non-affected participants. Thirdly, although we used the random number table method to select the respondents and telephoned them to participate in our research, the proportion of women was significantly higher than that of men, which may relate to China’s social reality that women are often doing housework at home and have more free time to take part in the social survey.

## 5. Conclusions

In this study, we found that among people using healthcare services, the mode of services, care provider, distance to practice and cost should be considered in priority-setting decisions. The government should strengthen the curative service capability in primary health facilities and give full play to the role of TCM in the prevention and treatment of severe NCDs. It is worth noting that health service utilization is a complex behavior affected by many factors, and the preferences of individuals may change as a function of their health status. A single intervention is unlikely to be sufficient or successful to meet the needs of residents with severe NCDs. Effective interventions must be combined in different policy intervention packages, and these interventions must be matched with the preferences and expectations of residents with severe NCDs.

## Figures and Tables

**Table 1:** Discrete choice experiment attributes and attribute levels.

Attributes	Levels	Definition
Mode of service	Traditional Chinese medicine (TCM) services Western medicine (WM) services Integrated TCM and WM services	The way of treatment for non-communicable diseases (NCDs).
Cost (CNY ^1^)	100 200 300	The average expense that individuals paid each time in medical institutions.
Distance to practice	≤30 min >30 min	Travel time to medical institutions by the most convenient transportation.
Care provider	Expert General practitioner (GP)	When registering, individuals choose the “Expert” or “GP”.

Notes: ^1^ The average annual exchange rate between USD and CNY in 2018 was: USD 1 = CNY 6.616 [33].

**Table 2:** Example of a choice question.

Which Option Would You Choose?		
	Option 1	Option 2
Mode of service	Western medicine services	Traditional Chinese medicine services
Care provider	Expert	General practitioner
Distance to practice	>30 min	≤30 min
Cost (CNY)	100	200
Tick one box only		

**Table 3:** Characteristics of respondents.

Characteristics of Respondents	Full Sample n = 2160	Analysis Sample: n = 2019 (Who Passed the Consistency Test)	Excluded Sample: n = 105 (Who Failed the Consistency Test)	χ^2^ (*p*-Value)
	n (%)	n (%)	n (%)	
**Sex**				2.37 (0.158)
Male	734 (33.98)	659 (32.60)	75 (53.19)	
Educational level				4.98 (0.274)
Elementary school and below	481 (22.65)	432 (21.40)	49 (46.67)	
Middle school	900 (42.37)	884 (43.80)	16 (15.24)	
**Occupation**				1.35 (0.659)
Working	1135 (53.44)	1071 (53.00)	64 (60.95)	
**Have medical insurance**				0.83(0.846)
Yes	1953 (91.95)	1857 (92.00)	96 (91.43)	
**Age/year (mean ± SD)**	48.13 (18.23)	47.50 (18.11)	48.45 (18.36)	
**Monthly income CNY (mean ± SD)**	5532 (869.24)	5503.59 (865.18)	5524.36 (848.15)	

**Table 4:** Results of mixed logit regression analysis and WTP.

Attribute Levels	β (SE)	SD (SE)	WTP
**Mode of service** (reference level, traditional Chinese medicine services)			
Western medicine services	0.394(0.025) *	1.482(0.045) *	−382.15
Integrated TCM and WM services	0.485(0.025) *	1.624(0.041) *	−470.54
**Distance to practice** (reference level, ≤30 min)			
>30 min	−0.071(0.018) *	1.073(0.019) *	68.2
**Care provider** (reference level, GP)			
Expert	0.847(0.018) *	2.33(0.043) *	−822.51
**Cost (CNY)**	−0.001(0.016) *	0.904(0.015) *	
**Log likelihood**	−9638.80		
**Respondents, n**	2019		
**Observations, n**	32,304		

Notes: * *p <* 0.01. SE: standard error. SD: standard deviation. WTP: willingness to pay.

## Abbreviations

DCE	Discrete choice experiment
WTP	Willingness to pay
WM	Western medicine
TCM	Traditional Chinese medicine
NCDs	Non-communicable chronic diseases
GP	General practitioner
SE	Standard error
SD	Standard deviation
